# Recombination and Genetic Diversity Analysis of Porcine Reproductive and Respiratory Syndrome 1 Nonstructural Protein 2 Genes in China

**DOI:** 10.3390/genes16050507

**Published:** 2025-04-28

**Authors:** Chen Lv, Baoyi Guan, Jiankun Pang, Weili Kong, Ruining Wang, Lin Wang, Mengmeng Zhao, Hang Zhang

**Affiliations:** 1 Guangdong Provincial Key Laboratory of Animal Molecular Design and Precise Breeding, School of Animal Science and Technology, Foshan University, Foshan 528225, China; lc0214fosu@163.com (C.L.); 20220420207@stu.fosu.edu.cn (B.G.); 15118664620@163.com (J.P.); 2 Gladstone Institutes of Virology and Immunology, University of California, San Francisco, CA 94158, USA; weili.kong@gladstone.ucsf.edu; 3 College of Veterinary Medicine, Henan University of Animal Husbandry and Economy, Zhengzhou 450046, China; 80882@hnuahe.edu.cn; 4 Institute of Cancer Sciences, University of Glasgow, Glasgow G12 8QQ, UK; lin.wang@glasgow.ac.uk

**Keywords:** genetic analysis, recombination, *NSP2* gene, China, PRRSV

## Abstract

Background: Porcine reproductive and respiratory syndrome (PRRS) has been present in China for about 30 years, and because of the high mutability of PRRSV, it causes huge economic losses to pig enterprises every year. PRRSV-2 is widely prevalent in China, and the detection rate of PRRSV-1 is also on the rise. Nonstructural protein 2 (NSP2) is a highly variable protein with multiple biological functions, such as PRRSV replication, which plays an important role in understanding PRRSV variation and epidemic alerts. Objectives: The epidemic characteristics and recombination of PRRSV-1 *NSP2* are still unknown. The purpose of this study is to study the epidemic characteristics of PRRSV-1 *NSP2* and lay a foundation for the prevention and control of PRRSV-1. Methods: In this study, we collected several PRRSV-1 and PRRSV-2 *NSP2* gene sequences for gene sequence and recombination analyses, aiming to analyze the recombination pattern and genetic variation in the PRRSV-1 *NSP2* genes in China. Results: The genetic similarity results showed that the 69 PRRSV-1 *NSP2* gene sequences collected in this study showed nucleotide similarity ranging from 67.3% to 100.0% and amino acid similarity ranging from 64.3% to 100.0%. Amino acid sequence comparison showed that PRRSV-1 had more amino acid deletion or substitution sites than PRRSV-2. NSP2 also contains special amino acid regions such as the highly immunogenic region. PRRSV-1 can be categorized into four strains, NMEU09-1-like, BJEU06-1-like, HKEU-16-like and Amervac-like isolates, and are at different positions in the ML and NJ phylogenetic trees. In the ninety selected PRRSVs, six recombination events were detected using recombination analysis, two of which occurred in Chinese PRRSV-1 strains. Therefore, sequence analysis of *NSP2* helps us to understand the prevalence and variation in PRRSV-1 in China over the past two decades and provides a theoretical basis for studying the epidemiology and evolution of *NSP2*.

## 1. Introduction

Porcine reproductive and respiratory syndrome (PRRS), also known as “pig blue ear disease”, is caused by the PRRS virus (PRRSV) and is a highly contagious disease. China experiences a high rate of losses in pig production, often attributed to reinfections from other diseases like pseudorabies, circovirus disease, and bacterial infections, including Escherichia coli in piglets [1]. In 1991 and 1992, the Lelystad virus (LV) strain was obtained in Europe, while the VR2332 strain was isolated from swine in the United States [2,3]. Currently, it is believed that two distinct species belonging to the *Betaarterivirus suid 1* and *Betaarterivirus suid 2* exist, also known as PRRSV-1 and PRRSV-2, respectively [4]. In 1996, PRRSV-2, also known as CH-1a, was first discovered in Harbin, China, with its complete genome sequence deposited in GenBank in the same year [5]. In 1997, an outbreak of PRRSV-1, also known as B13, was first reported in Beijing, China [6]. PRRSV-2 is mostly found in Asia and North America, in addition to PRRSV-1. PRRSV-2 is exclusively found in the southern part of the American continent, whereas PRRSV-1 is more common in Europe. Nonetheless, PRRSV-2 has also been observed in Western Europe. With the widespread spread of PRRSV in China, mutations and recombinations have occurred. These recombinations occur not only in wild strains but also between wild strains and modified live vaccines (MLV) and even between two different MLV vaccine strains [7,8]. The emergence of recombination phenomena has led to the emergence of new recombinant strains of PRRSV and the phenomenon of virulence reintroduction, which has made the clinical situation of preventing and controlling the PRRS outbreak even more severe and complicated.

PRRSV is an RNA virus with a positive-sense genome enclosed within an envelope. The genome is approximately 15 kb and consists of eleven open reading frames (ORFs) [9]. Specifically, the genome of Lelystad measures 15,111 bp, while the genome of VR2332 is slightly larger at 15,182 bp. ORF1a is responsible for translating at least ten nonstructural proteins (NSPs), including NSP1α/β, NSP1-6, NSP7α/β, and NSP8. Nonstructural protein 2 (NSP2) is the largest replicase cleavage product of PRRSV, which is about 2.9 kb and highly variable, and is the most variable protein in the PRRSV genome [10,11,12,13,14]. NSP2 contains six structural domains, including the N-terminal hypervariable region-I (HV-I), the cysteine protease PLP2 region, the structure of the peptidase C33, the 500–700 aa hypervariable region-II (HV-II) and the C-terminal hydrophobic transmembrane region (TM), in which the PLP2 functional domain has both cis- and trans-cleavage activity, cleaving its own peptide to produce multiple NSP2 subunits (NSP2a-f) or cleaving the region between NSP2 and NSP3 [13,15,16]. Among these, the NSP 2TF and NSP 2N proteins can increase the functional complexity of the NSP2 region of the viral replicase, and both share the N-terminal PLP2 structural domain with NSP2 [17,18]. In addition, HV-II is responsible for the lower amino acid similarity of NSP2 in both the same and different subtypes of strains compared to other more conserved proteins of PRRSV [19]. *NSP2* is often used together with the *GP5* gene as a subject of molecular epidemiological investigations as well as mutational analyses of PRRSV and plays an important role in understanding viral variation and outbreak alerts.

PRRSV-1 ORF5 sequences from GenBank were analyzed by Shi et al., who identified three subtypes: Subtype I (Russian, Global), Subtype II (Belarus, Lithuania, Russian), and Subtype III [20]. Initially discovered in Western Europe, Subtype I of PRRSV-1 has subsequently spread to the Americas and Asia, and it can be further categorized into 12 branches. Meanwhile, Subtype II and III are primarily found in Eastern Europe and Russia. The majority of PRRSV-1 isolates in China belong to Global Subtype I. There are four subgroups of PRRSV-1. Chen et al. [21] categorized PRRSV-1 into NMEU09-1-like, BJEU06-1-like, Amervac-like, and HKEU-16-like subtypes. China has a high incidence of PRRSV-2, which has led to most studies being focused on investigating PRRSV-2. In recent years, there has been an increase in the incidence of PRRSV-1, thus emphasizing the need for further research on the function of *NSP2* in PRRSV-1 strain [8,22,23]. This study aimed to understand recombination events and genetic diversity of *NSP2* in PRRSV-1 strains. To compare nucleotide and amino acid similarities, multiple amino acid sequence alignments were performed, phylogenetic relationships were examined, and recombination events were analyzed. These results will contribute to monitoring of the epidemic patterns of PRRSV-1.

## 2. Materials and Methods

### 2.1. Strain Collection

Sixty-nine PRRSV-1 *NSP2* strains (twenty-five from China and forty-four from overseas) and twenty-one PRRSV-2 strains were carefully selected from the NCBI website (Table 1). In this study, PRRSV-1 strains that have appeared in China and abroad, including classical PRRSV-1 strains and strains that have appeared in recent years, were included in order to comprehensively analyze the genetic variation in the PRRSV-1 *NSP2* gene over time and across multiple strains and to help differentiate between domestic and foreign PRRSV-1 strains. In addition, some representative PRRSV-2 strains were selected for comparative analysis to determine the genetic evolutionary differences between the two genotypes.

### 2.2. Sequence Analysis of the PRRSV-1 NSP2

We examined sixty-nine PRRSV-1 *NSP2* sequences for nucleotide and amino acid similarities. The CDs of 69 PRRSV-1 *NSP2* strains downloaded from the NCBI website (https://www.ncbi.nlm.nih.gov/ (accessed on 4 April 2025)) were processed into nucleotide sequences using the EditSeq function in the DNAStar software (Version 7.0, Madison, WI, USA), where the nucleotide sequences were further processed into amino acid sequences, and subsequently all sequences were aligned using the Clustal W method in the MegAlign function, and the final output of the nucleotide and amino acid similarity comparison results. In addition, the *NSP2* nucleotide sequences of 34 PRRSV-1 and 6 PRRSV-2 strains were selected, and these 34 PPRSV-1 strains are strains from different countries and times, including classical and vaccine strains. Then, they were translated into amino acid sequences in MegAlign, and the amino acid sequences were also processed using the Clustal W method to output the amino acid sequence comparison results. Finally, some regions were labeled and annotated using Adobe Photoshop (version 2022).

### 2.3. Phylogenetic Analysis

Phylogenetic evaluation of ninety PRRSV *NSP2* sequences (Table 1) was performed using the maximum likelihood (ML) method and the neighbor-joining (NJ) method with 1000 bootstrap replicates of MEGA software (Version 11, Mega Limited, Auckland, New Zealand). Subsequently, the sequences were annotated using a web-based tool called The Interactive Tree of Life (https://itol.embl.de (accessed on 15 April 2025)).

### 2.4. Recombination Analysis

Seven methods (Chimaera, BootScan, 3Seq, GeneConv, SiScan, MaxChi, and RDP) of RDP software (version 4.101) were used to identify potential recombination events. Recombination events that were detected by at least six methods with a *p*-value of less than 0.05 were significant. In addition, recombination events identified through SimPlot (version 3.5.1) were subjected to validation.

## 3. Results

### 3.1. Analysis of Nucleotide and Amino Acid Similarity of the PRRSV-1 NSP2

In this research, twenty-five Chinese and forty-four overseas PRRSV-1 *NSP2* sequences were analyzed to assess their nucleotide similarity (Table 2 and Table S1). The findings revealed that the nucleotide similarity of the PRRSV-1 *NSP2* was 67.3–100.0%. The similarity between Lena and lena (100.0%) was the highest, whereas that between Tyu16 and BE_08V156 was 67.3%. The amino acid similarity of PRRSV-1 NSP2 was between 64.3% and 100.0%. Similarities between lena and Lena (100.0%) were the highest, whereas similarity between Tyu16 and 15HEN1_EU (64.3%) was the lowest.

### 3.2. Amino Acid Sequence Alignment

**Figure 1:**
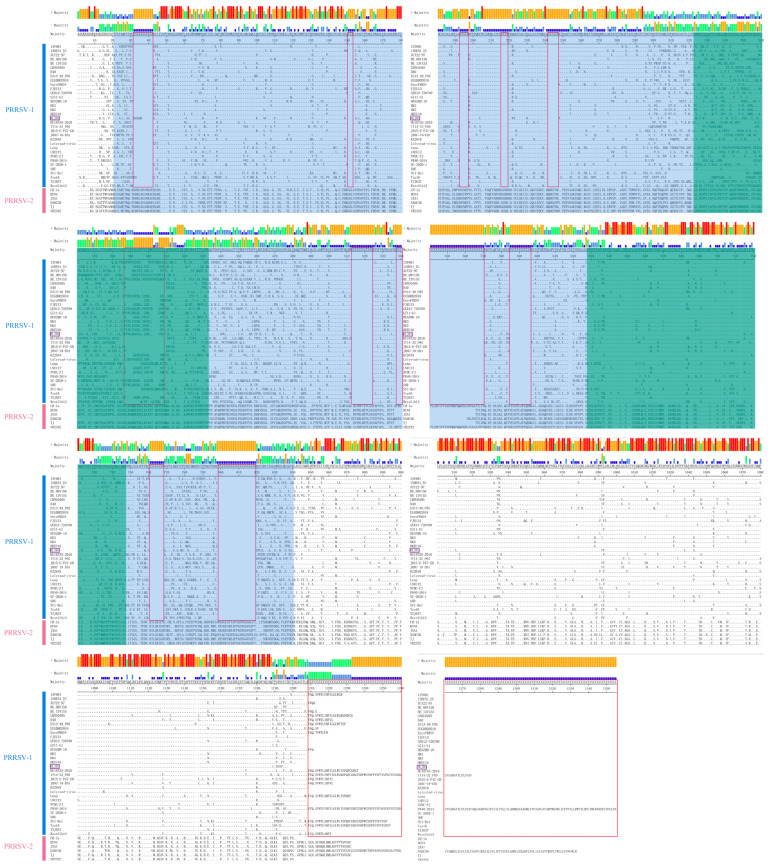
NSP2 amino acid sequence alignment effects of representative PRRSV strains. The colored bars in the plot range from blue to red, with high to low levels of variation at that amino acid site. Amino acid similarity sites are denoted by “.”. Red areas represent PRRSV-1 NSP2 amino acid deletion sites, the blue background represents a highly immunogenic region that NSP2 has, the green background represents a region of NSP2 related to PRRSV replication, and the recombinant strains are shown in purple boxes.

To evaluate the diversity of PRRSV-1 and PRRSV-2 NSP2 amino acid sequences, thirty-four PRRSV-1 NSP2 and six PRRSV-2 NSP2 sequences were selected for multiple sequence alignments (Figure 1). The results confirmed a higher number of amino acid deletion sites in PRRSV-1 NSP2 than in PRRSV-2, indicating lower similarity in PRRSV-1 NSP2. These deletion sites were primarily located within amino acid positions 32–42, 151–153, 193–197, 216–219, 242–247, 387–408, 513–524, 538–570, 585–596, 761–768 and 799–819, and there are a number of scattered positive sites. Furthermore, areas with higher mutation rates were largely centered at positions 32–42, 242–247, 513–524, 538–570, and 799–819 of the amino acid sequences. The variation in NSP2 among PRRSV-1 did not display a specific pattern, and deletion of NSP2 was the highest at the 538–570 site. In addition, mutations and deletions of amino acids similarly occur in the highly immunogenic region of NSP2 and in the region associated with PRRSV replication.

### 3.3. Analysis of Phylogenetic Analysis

**Figure 2:**
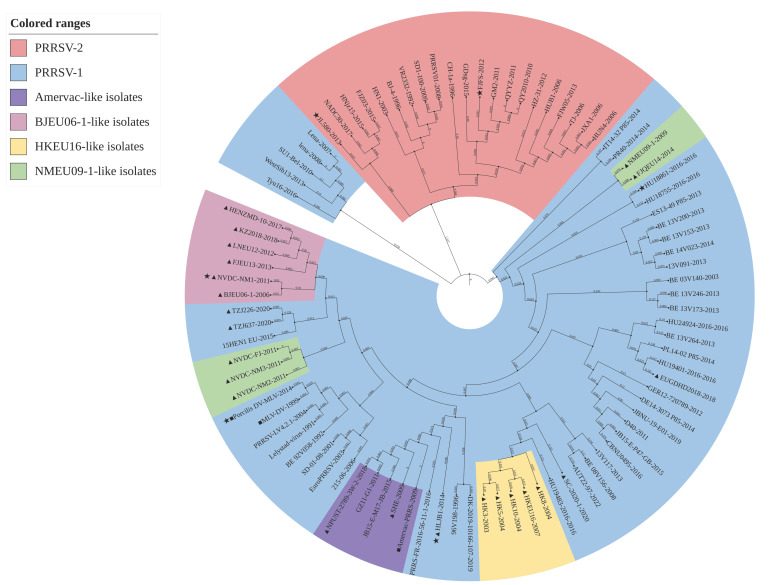
Phylogenetic evaluation of the PRRSV *NSP2* gene was performed using the ML method. Sixty-nine PRRSV-1 and twenty-one PRRSV-2 strains were chosen for the analysis. PRRSV-2 strains are highlighted using a red background color, while PRRSV-1 strains are highlighted using a blue background color. Chinese PRRSV-1 strains are represented by triangles (▲), PRRSV-1 vaccine strains are indicated with squares (■), recombinant strains are represented by triangles (★), and the numbers represent the branch lengths.

**Figure 3:**
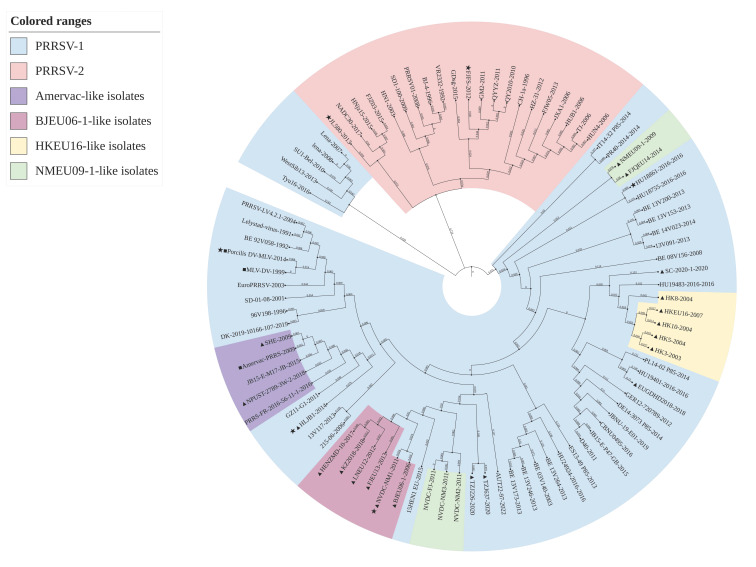
Phylogenetic evaluation of the PRRSV *NSP2* gene was performed using the NJ method. A total of sixty-nine PRRSV-1 strains and twenty-one PRRSV-2 strains were chosen for the analysis. PRRSV-2 strains are highlighted using a red background color, while PRRSV-1 strains are highlighted using a blue background color. Chinese PRRSV-1 strains are represented by triangles (▲), PRRSV-1 vaccine strains are indicated with squares (■), recombinant strains are represented by triangles (★), and the numbers represent the branch lengths.

Phylogenetic tree analysis showed that the 90 strains used in the treatment could be divided into two branches, PRRSV-1 and PRRSV-2 (Figure 2 and Figure 3). PRRSV-1 could be further divided into two branches, and the larger branch could be divided into Amervac-like isolates, BJEU06-1-like isolates, HKEU16-like isolates and NMEU09-1-like isolates. In the ML phylogenetic tree, among the NMEU09-1-like isolates, the FJQEU14 and NMEU09-1 strains were genetically closer to PRRSV-2, and the BJEU06-1-like isolates were the most genetically related to PRRSV-1. In the NJ phylogenetic tree, Amervac-like isolates were most closely genetically related to PRRSV-1, and BJEU06-1-like isolates and NMEU09-1-like isolates were more closely genetically related. Notably, the Amervac PRRS vaccine strain showed a close genetic relationship with the SHE strains in both results, and both the European representative LV strain and PRRSV-LV4.2.1 were within the same evolutionary branch.

### 3.4. Recombinant Analysis

**Figure 4:**
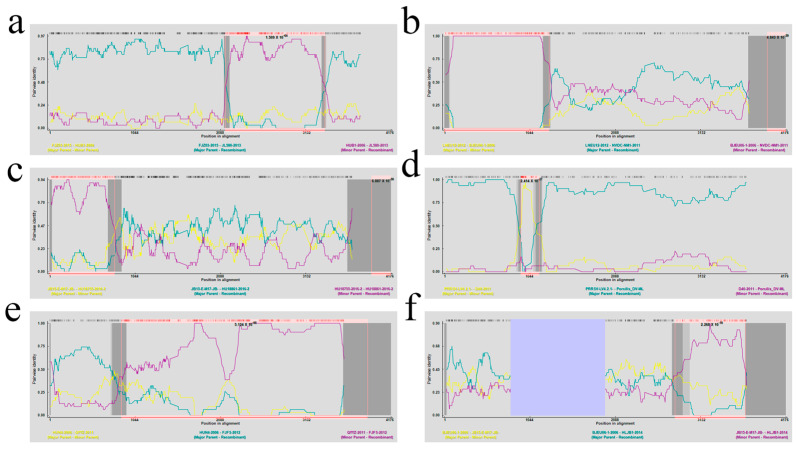
Potential recombination events in the *NSP2* gene as detected using RDP (Version 4.101). The horizontal axis indicates the position in alignment, while the vertical axis illustrates pairwise identity. Panels (**a**−**f**) correspond to the RDP software predictions for each recombinant strain in Table 3, respectively.

**Figure 5:**
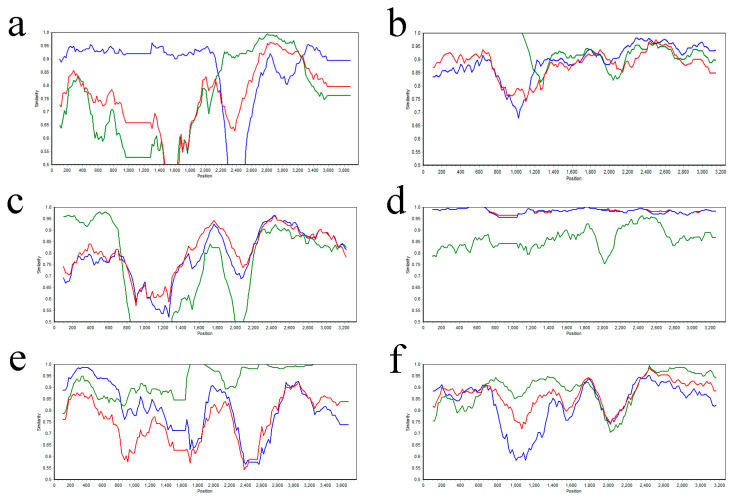
Verification of recombination events in the *NSP2* gene through SimPlot (Version 3.5.1). The horizontal axis denotes the position, and the vertical axis signifies the percentage of permuted trees. Panels (**a**–**f**) correspond to the validation results of SimPlot software for recombination events for each recombinant strain in Figure 4, respectively. The main parental strain is depicted by the blue line, the minor parental strain by the green line, and the control strain by the red line.

This study aimed to improve our comprehension of the genetic development of PRRSV-1 *NSP2* through the analysis of recombination events in *NSP2* sequences obtained from a sample of ninety PRRSV strains. We conducted the analysis of the collected sequences using RDP software (version 4.101), revealing six possible occurrences of recombination. These six instances of recombination were substantiated by more than five distinct algorithms, suggesting their strong credibility and statistical significance (*p* < 0.05) (Table 3 and Figure 4). A total of four sets of recombination events were predicted for the PRRSV-1 strain. In the first recombination event, NVDC-NM1, which was the recombinant strain, contained LNEU12 as the main parental strain and BJEU06-1-2006 as the minor parental strain. Similarly, in the second recombination event, HU18861-2016, which was the recombinant strain, contained JB15-E-M17-JB as the main parental strain and HU18755-2016 as the minor parental strain. In the third recombination event, Porcilis_DV-MLV, which was the recombinant strain, contained PRRSV-LV4.2.1 as the main parental strain and D40 as the minor parental strain. Finally, in the fourth recombination event, HLJB1, which was the recombinant strain, contained BJEU06-1 as the main parental strain and JB15-E-M17-JB as the minor parental strain. In addition, recombination events 1 and 5 were recombinations that occurred in PRRSV-2. JL580 and FJFS strains were the two strains that underwent recombination, FJZ03 and HUN4 strains were their main parental strains, and HUB1 and QYYZ strains were their minor parental strains, respectively. We utilized SimPlot (Version 3.5.1) to conduct our assessment of the six recombination events, confirming their validity (Figure 5). The results showed that recombination events 1, 2, 3, and 6 were verified as recombination events by SimPlot, which contained one recombination event within PRRSV-2 and three within PRRSV-1.

## 4. Discussion

Since its emergence, PRRS has caused great economic losses to the global pig industry due to its highly infectious nature, and the emergence of new mutant strains with the continuous spread and mutation of PRRSV has also brought great pressure to the clinical prevention and control of PPRS [3,24]. PRRSV-1 and PRRSV-2 outbreaks in China occurred in 1997 and 1996, respectively [5,6]. Now PRRSV-1 has been sporadically endemic and is relatively mildly pathogenic, causing mainly reproductive problems in pigs and occasional cases of respiratory distress, including fever [25]. Consequently, the majority of studies have focused on PRRSV-2. Nevertheless, in recent years, the occurrence of PRRSV-1 has risen in several areas of China, which have reported the identification of PRRSV-1 [26]. The emergence of PRRSV-1 has been reported in major importing countries of Chinese breeding pigs, and the emergence of PRRSV-1 has been reported in all of these countries [27,28]; however, China prohibits the use of PRRSV-1 modified live vaccine, so it is highly likely that PRRSV-1 in China originates from imported breeding pigs. Yu et al. [29] showed that PRRSV-1 strains first entered China as the LV-like PRRSV strain by 2006 and then formed an independent branch in the spread of the virus, and the most recent introduction occurred before 2009, which may be related to the trade of pigs between China and Denmark. In recent years, PRRSV-1 has been reported in several provinces and regions of China. Therefore, conducting epidemiologic studies of PRRS and monitoring genetic variation in PRRSV-1 are essential for the prevention and control of PRRS epidemics [26,29,30].

The NSP2 protein, the most mutated protein of PRRSV, has six structural domains with different functions and plays important functions in PRRSV assembly, transcription, replication, and regulation of immune responses through interactions with PRRSV self and host proteins [15,17,31]. Replication of the *NSP2* gene occurs in a bilayer membrane vesicle located in the cytoplasm close to the nucleus. This region is crucial for the replication and transcription of PRRSV. In addition, specific regions of the NSP2 protein (amino acid regions 323–433, 628–747, and 727–747) annotated in Figure 1 have a critical role in PRRSV replication. Previous research has demonstrated that transfecting PRRSV NSP2 plasmid into cells and establishing a stable NSP2 cell line can be used to measure the virus concentration. These experiments showed that NSP2 can promote PRRSV proliferation [32], suggesting that the 727–747 amino acid region of NSP2 may be related to the replication capacity of PRRSV [33]. Wang et al. [32] reported that NSP2 enhances the replication of PRRSV in Marc-145 cells. Conversely, when NSP2 is interfered with in Marc-145 cells, it leads to the inhibition of PRRSV replication. Further investigation of the mechanism by which NSP2 promotes PRRSV replication revealed that the NSP2 C-terminal region is independent of PRRSV replication [34]. However, the amino acid regions 323–433 and 628–747 within NSP2 play a vital role in the PRRSV replication process [35]. Liu et al. [36] further proved that deletion of the 628–727 amino acid region of NSP2 in the TJM strain could inhibit PRRSV proliferation by truncating the transient expression of the protein.

A distinctive feature of PRRSV is the excessive number of mutations, especially in the *NSP2* gene, which displays various types of mutations such as deletion, recombination, and insertion, resulting in noticeable variations in the NSP2 proteins [37]. The similarity between the LV and VR2332 strains was only 40.0%. The CH-1a strain of PRRSV-2, first discovered in China, shares 80.0% similarity with *NSP2* of the VR2332 strain from the United States. According to Zhang et al. [38], the KZ2018 strain, which is also known as PRRSV-1, shares approximately 88.6% similarity with the LV strain and has 81.9–90.8% similarity with other PRRSV-1 strains found in China. Shen et al. [39] studied the entire nucleotide sequence of the genomic RNA of vaccine strain SP. These findings revealed that the vaccine strain SP exhibited only 78.0% nucleotide sequence similarity with the European LV strain. The most significant variation was observed in the C-terminus of NSP2, wherein 36 and 155 amino acids were inserted. This insertion sequence did not show any similarity with equivalent arterial virus proteins. The notable differences in NSP2 among various PRRSV isolates indicate its potential utility as a marker to distinguish between PRRSV genotypes. Furthermore, NSP2 of the PRRSV-1 KNU-07 strain, which was initially isolated in South Korea in 2009, exhibited a deletion of 60 amino acids [40]. Consequently, *NSP2* is commonly employed as a target gene to analyze the evolution of PRRSV. In this study, 25 Chinese PRRSV-1 *NSP2* sequences and 44 foreign PRRSV-1 *NSP2* sequences were obtained from the NCBI database and analyzed for genetic similarity. The findings indicated that the amino acid and nucleotide resemblance varied from 64.3 to 100.0% and 67.3 to 100.0%, respectively. Zhang et al. [41] reported that the amino acid and nucleotide similarities in PRRSV-2 NSP2 ranged from 72.5 to 99.8% and 63.9 to 99.4%, respectively. The degree of variation in the amino acid sequence of PRRSV-1 NSP2 was higher than that of PRRSV-2 NSP2, but the degree of variation in nucleotide sequence was comparable to that of PRRSV-2 *NSP2*, which may be attributed to the accumulation of nonsynonymous mutations in some key loci of PRRSV-1 strains under the positive selection pressure and the occurrence of adaptive changes in genes in order to adapt to the epidemiology in China. In addition, the diversity of sample sources is also one of the important reasons affecting the results of gene sequence analysis, and samples from different countries and regions may have different genotypes and genealogical relationships under them. In our investigation, the analysis of amino acid sites between PRRSV-1 and PRRSV-2 NSP2 by aligning thirty-four PRRSV-1 and six PRRSV-2 *NSP2* sequences indicated that PRRSV-1 NSP2 showed a higher number of deletion and substitution sites than PRRSV-2. In addition, PRRSV NSP2 has a highly immunogenic region, 21-840aa [42,43], which has multiple B-cell linear epitopes, and different parts of the region showed mutations and deletions of the locus. The results of the amino acid variation (Figure 1) showed that different strains had different degrees of variability in the hypervariable and hyperimmunogenic regions, which could be an adaptive evolution of PRRSV to facilitate immune escape and adaptation of the virus itself for better transmission. It can be seen that the deletion and mutation of amino acids in the NSP2 region of PRRSV-1 pose an obstacle to the effective use of existing vaccines but also provide new directions for the design of new vaccines.

Recombination and mutation are important factors in the evolution of RNA viruses, and recombinant viruses can inherit adaptive features from parental strains to enhance host adaptation and transmission. PRRSV exhibits significant genetic diversity due to its high-frequency mutation and recombination properties, leading to new strains that break through the efficacy of existing vaccine protection [44,45]. The prevalence of PRRSV-1 strains in China may be associated with vaccinated breeding pigs, while factors such as quarantine loopholes and latent infections contribute to the genetic recombination of PRRSV-1, and some of the recombinant strains have demonstrated enhanced pathogenicity and transmission capacity [46], which has resulted in a double blow to the Chinese swine industry from both PRRSV-1 and PRRSV-2. Analyzing the recombination events of PRRSV-1 strains in China and abroad is helpful in revealing its evolutionary pattern in China. When new strains are isolated in the clinic, the combination of phylogenetic tree analysis can rapidly trace back the parental strains and provide a theoretical basis for vaccine selection. The results of our study show that the 90 strains used in the phylogenetic trees constructed based on the ML and NJ methods could be categorized into two genotypes, PRRSV-1 and PRRSV-2, but the division of the similar strains within PRRSV-1 was different in different evolutionary trees. The NMEU09-1-like and BJEU06-1-like strains were genetically closer in the phylogenetic trees constructed by both the ML and NJ methods, and the NMEU09-1-like strain could be similarly divided into two parts. It is worth noting that the HLJB1 strain belonged to the BJEU06-1-like lineage in Chen et al.’s [21] study, whereas it did not belong to any of the sub-lineages in the present study, but both were genetically close to the Amervac-like lineage, which may be due to factors such as algorithmic updating resulting from the iteration of the analysis software. In addition, HKEU-16-like strains were more independent in both phylogenetic trees. However, the results of the analysis of the complete genome sequences varied. Specifically, BJEU06-1-like was more closely genetically related to NMEU09-1-like and Amervac-like strains. This finding further confirms the close genetic linkage between BJEU06-1-like and NMEU09-1-like strains [8]. The reason for this difference may also lie in the significant variation observed in the PRRSV-1 *NSP2* gene. Therefore, based on the results of this study, it is possible to classify PRRSV-1 solely on the basis of the *NSP2* gene. In our study, six potential cases of recombination were identified. Notably, two of the recombination cases were observed in China, and both involved Chinese parental strains, and all six recombinant strains belonged to or were very close to four subspectrums, including Amervac-like strains. However, Chen et al. [47] indicated that the formation of the HLJB1 isolate resulted from a recombination occurrence between the Amervac vaccine variant and the BJEU06-1 strain. The discrepancy in these discoveries might have arisen because Chen et al. conducted an exhaustive analysis of the complete sequence for identifying recombination cases, while our research solely concentrated on the *NSP2* sequence.

In China, vaccination plays a key role in the prevention and control of PRRS, with a major focus on domestically produced vaccines against PRRSV-2 [48]. For PRRSV-1, there are many international vaccines, such as Suvaxyn PRRS MLV (Zoetis, Belgium, USA) and ReproCyc PRRS EU (Boehringer Ingelheim, Ingelheim am Rhein, Germany, etc.) The PRRSV NSP2 region is particularly important in vaccine research because it tolerates deletion of the deletion fragment and maintains stable expression of the inserted genes. Currently, despite the extensive understanding of the pathogenesis, molecular epidemiology, and immune response of PRRSV, timely monitoring of genetic changes in PRRSV could provide a valuable reference for the effective management of PRRS in China in the coming years.

## 5. Conclusions

From the first appearance of PRRSV-1 in China in 1997 to 2022, the prevalence of PRRSV-1 in China has been more sporadic than that of PRRSV-2, but in recent years, its prevalence has been on the rise. NSP2 is a protein with a high degree of PRRSV variability, which can be involved in various aspects of PRRSV replication and immunomodulation. The PRRSV-1 strains that emerged in China were categorized into four different sub-lineages: NMEU09-1-like, HKEU-16-like, BJEU06-1-like, and Amervac-like, and the PRRSV-1 *NSP2* sequences showed a greater diversity of substitutions, deletions, and mutations than the PRRSV-2 *NSP2* sequences. In addition, recombination analysis showed that recombination occurred between both PRRSV-1 and PRRSV-2 strains. By analyzing the gene sequence of PRRSV-1 *NSP2*, we can visualize its genetic evolution and mutation trends during the more than two decades of its prevalence in China and provide a theoretical basis to guide the further study of the biological function of NSP2 and the development of a novel vaccine.

## Figures and Tables

**Table 1:** Information on the reference sequence of PRRSV *NSP2*.

Year	Aera	Strain	GenBank Accession Number	Type
1991	Netherlands	Lelystad virus	M96262	PRRSV-1
1992	Belgium	BE_92V058	MW448197	PRRSV-1
1996	Belgium	96V198	MK876228	PRRSV-1
1999	Netherlands	MLV-DV	KJ127878	PRRSV-1
2001	USA	SD-01-08	DQ489311	PRRSV-1
2003	China	HK3	KF287129	PRRSV-1
2003	USA	EuroPRRSV	AY366525	PRRSV-1
2003	Belgium	BE_03V140	MW053394	PRRSV-1
2004	China	HK5	KF287130	PRRSV-1
2004	China	HK8	KF287128	PRRSV-1
2004	China	HK10	KF287131	PRRSV-1
2004	Netherlands	PRRSV LV4.2.1	AY588319	PRRSV-1
2006	China	BJEU06-1	GU047344	PRRSV-1
2006	United Kingdom	215-06	OP047897	PRRSV-1
2007	China	HKEU16	EU076704	PRRSV-1
2007	Belarus	Lena	JF802085	PRRSV-1
2008	Belgium	BE_08V156	MW053397	PRRSV-1
2008	Belarus	lena	JF802085	PRRSV-1
2009	China	SHE	GQ461593	PRRSV-1
2009	China	NMEU09-1	GU047345	PRRSV-1
2009	Spain	Amervac PRRS	GU067771	PRRSV-1
2010	Belarus	SU1-Bel	KP889243	PRRSV-1
2011	China	GZ11-G1	KF001144	PRRSV-1
2011	China	NVDC-FJ	KC492506	PRRSV-1
2011	China	NVDC-NM1	JX187609	PRRSV-1
2011	China	NVDC-NM2	KC492504	PRRSV-1
2011	China	NVDC-NM3	KC492505	PRRSV-1
2011	South Korea	D40	MZ287330	PRRSV-1
2012	China	LNEU12	KM196101	PRRSV-1
2012	Germany	GER12-720789	OP529852	PRRSV-1
2013	China	FJEU13	KP860912	PRRSV-1
2013	Russia	WestSib13	KX668221	PRRSV-1
2013	Belgium	13V117	KT159249	PRRSV-1
2013	Belgium	BE_13V246	MW053396	PRRSV-1
2013	Belgium	BE_13V173	MW053395	PRRSV-1
2013	Belgium	13V091	KT159248	PRRSV-1
2013	Belgium	BE_13V200	MW053399	PRRSV-1
2013	Belgium	BE_13V153	MW053398	PRRSV-1
2013	Spain	ES13-49_P85	MK024325	PRRSV-1
2013	Belgium	BE_13V264	MW053400	PRRSV-1
2014	China	FJQEU14	KP860913	PRRSV-1
2014	China	HLJB1	KT224385	PRRSV-1
2014	Germany	DE14-3073_P85	MK024324	PRRSV-1
2014	Denmark	Porcilis_DV-MLV	MT311646	PRRSV-1
2014	Belgium	BE_14V023	MW053401	PRRSV-1
2014	Poland	PL14-02_P85	MK024327	PRRSV-1
2014	Italy	PR40/2014	MF346695	PRRSV-1
2014	Italy	IT14-32_P85	MK024326	PRRSV-1
2015	China	15HEN1_EU	KX967492	PRRSV-1
2015	South Korea	JB15-E-M17-JB	MZ287329	PRRSV-1
2015	South Korea	JB15-E-P47-GB	MZ287328	PRRSV-1
2016	South Korea	CBNU0495	MZ287327	PRRSV-1
2016	France	PRRS-FR-2016-56-11-1	MH018883	PRRSV-1
2016	Hungary	HU19401/2016	MH463457	PRRSV-1
2016	Hungary	HU24924/2016	MH463459	PRRSV-1
2016	Hungary	HU19483/2016	MH463458	PRRSV-1
2016	Hungary	HU18861/2016	MH463456	PRRSV-1
2016	Hungary	HU18755/2016	MH463455	PRRSV-1
2016	Russia	Tyu16	MT008024	PRRSV-1
2017	China	HENZMD-10	KY363382	PRRSV-1
2018	China	KZ2018	MN550991	PRRSV-1
2018	China	EUGDHD2018	MK639926	PRRSV-1
2018	China	NPUST-2789-3W-2	MN242825	PRRSV-1
2019	South Korea	JBNU-19-E01	MW847781	PRRSV-1
2019	Denmark	DK-2019-10166-107	MN603982	PRRSV-1
2020	China	SC-2020-1	MW115431	PRRSV-1
2020	China	TZJ226	OP566682	PRRSV-1
2020	China	TZJ637	OP566683	PRRSV-1
2022	Austria	AUT22-97	OP627116	PRRSV-1
1992	USA	VR2332	EF536003.1	PRRSV-2
1996	China	BJ-4	AF331831	PRRSV-2
1996	China	CH-1a	AY032626	PRRSV-2
2003	China	HN1	AY457635.1	PRRSV-2
2006	China	TJ	EU860248	PRRSV-2
2006	China	HUB1	EF075945	PRRSV-2
2006	China	JXA1	EF112445	PRRSV-2
2006	China	HUN4	EF635006.1	PRRSV-2
2008	China	PRRSV01	FJ175687	PRRSV-2
2009	China	SD1-100	GQ914997	PRRSV-2
2010	China	QY2010	JQ743666	PRRSV-2
2011	China	GM2	JN662424	PRRSV-2
2011	China	QYYZ	JQ308798	PRRSV-2
2012	China	HZ-31	KC445138	PRRSV-2
2012	China	FJFS	KP998476	PRRSV-2
2013	China	JL580	KR706343.1	PRRSV-2
2013	China	FJW05	KP860911	PRRSV-2
2015	China	GDsg	KX621003	PRRSV-2
2015	China	HNjZ15	KT945017	PRRSV-2
2015	China	FJZ03	KP860909	PRRSV-2
2017	China	NADC30	MH500776.1	PRRSV-2

**Table 2:** Evaluation of nucleotide and amino acid similarities among sixty-nine PRRSV-1 NSP2.

	Tyu16	Lena
15HEN1_EU	^a^ 64.3 (aa)	
lena		^b^ 100 (nt, aa)
BE_08V156	^a^ 67.3 (nt)	

^a^ indicates the lowest similarity. ^b^ indicates the highest similarity. nt indicates the nucleotide, aa indicates the amino acid.

**Table 3:** Recombination analysis of the PRRSV *NSP2* gene using RDP.

Recombination Event	Recombinant Strain		Recombinant Breakpoint	Recombination Analysis Method (*p*-Value)						
	Main Parental Strain	Minor Parental Strain		RDP	GENECONV	BootScan	MaxChi	Chimaera	SiScan	3seq
1	JL580		2134–2205 (3328–3386)	1.509 × 10^−64^	1.123 × 10^−54^	NS	4.298 × 10^−27^	2.053 × 10^−29^	1.630 × 10^−38^	1.020 × 10^−11^
	FJZ03	HUB1								
2	NVDC-NM1		3718–66 (1204–1301)	4.846 × 10^−30^	5.298 × 10^−26^	8.873 × 10^−26^	1.700 × 10^−20^	4.974 × 10^−16^	6.034 × 10^−34^	1.020 × 10^−11^
	LNEU12	BJEU06-1-2006								
3	HU18861-2016		3641–35 (713–884)	6.887 × 10^−30^	9.225 × 10^−29^	5.293 × 10^−32^	1.287 × 10^−6^	1.224 × 10^12^	1.179 × 10^−22^	1.020 × 10^−11^
	JB15-E-M17-JB	HU18755-2016								
4	Porcilis_DV-MLV		881–940 (1090–1198)	2.414 × 10^−27^	1.319 × 10^−27^	3.401 × 10^−12^	1.545 × 10^−10^	7.047 × 10^−10^	7.332 × 10^−8^	1.020 × 10^−11^
	PRRSV-LV4.2.1	D40								
5	FJFS		746–945 (3590–14)	5.124 × 10^−5^	6.492 × 10^−20^	2.196 × 10^−23^	1.345 × 10^−13^	1.414 × 10^−8^	1.543 × 10^−45^	3.633 × 10^−25^
	HUN4	QYYZ								
6	HLJB1		2783–3002 (3678–33)	2.268 × 10^−10^	5.446 × 10^−6^	1.729 × 10^−8^	5.043 × 10^−7^	3.227 × 10^−4^	4.590 × 10^−10^	5.884 × 10^−3^
	BJEU06-1	JB15-E-M17-JB								

## Data Availability

All datasets are available in the main manuscript. The dataset supporting the conclusions of this article is included within the article.

## Supplementary Materials

The following supporting information can be downloaded at https://www.mdpi.com/article/10.3390/genes16050507/s1, Figures S1 and S2: Amino acid sequence alignment of the PRRSV-1 NSP2; Table S1: Analysis of nucleotide and amino acid similarity of the PRRSV-1 NSP2.
